# PTF1a Activity in Enriched Posterior Foregut Endoderm, but Not Definitive Endoderm, Leads to Enhanced Pancreatic Differentiation in an* In Vitro* Mouse ESC-Based Model

**DOI:** 10.1155/2016/6939438

**Published:** 2016-01-06

**Authors:** Gopika G. Nair, Jon S. Odorico

**Affiliations:** ^1^Division of Transplantation, Department of Surgery, University of Wisconsin-Madison School of Medicine and Public Health, Madison, WI 53792, USA; ^2^Diabetes Research Center, University of California-San Francisco, San Francisco, CA 94143, USA

## Abstract

Transcription factors are tools repetitively used by the embryo to generate a variety of lineages. Hence, their context of activation is an important determinant of their ability to specifically trigger certain cell fates, but not others. The context is also consequential when considering directing differentiation of embryonic stem cells (ESCs). In this study, we sought to assess the context of pancreatic transcription factor 1a (PTF1a) activation in reference to its propancreatic effects in mouse ESCs (mESCs). We hypothesized that an enriched endodermal population would respond to PTF1a and trigger the pancreatic program more effectively than a spontaneously differentiated population. Using an* in vitro* model of pancreas development that we recently established, we found that inducing PTF1a in highly enriched definitive endoderm did not promote pancreatic differentiation but induction in more differentiated endoderm, specifically posterior foregut endoderm, did form pancreatic progenitors. These progenitors never underwent terminal differentiation to endocrine or acinar phenotype. However, a short 3D culture period, prior to PTF1a induction, led to the generation of monohormonal insulin^+^ cells and amylase-expressing cells. Our findings suggest that enriched posterior foregut endoderm is competent to respond to PTF1a's propancreatic activity; but a 3D culture environment is essential for terminal differentiation of pancreatic progenitors.

## 1. Introduction

ESCs hold great potential in regenerative medicine due to their unlimited ability to self-renew and differentiate to a repertoire of lineages and hence have been the focus of many differentiation studies to obtain transplantable cell types. For example, making functional *β* cells successfully promises a cure for Type 1 Diabetes. Definitive endoderm (DE) is the gastrulation-derived cell population that ultimately gives rise to the respiratory and digestive tract organs, including the pancreas. Therefore, efforts to generate functional *β* cells involve directed differentiation of ESCs to DE followed by stepwise differentiation to pancreatic cells, inspired by processes from normal pancreatic development.

Several studies have used TGF-*β* family molecules such as Activin A, Nodal, and BMP4 [1–6] or small molecules [7, 8] that mimic endogenous nodal signaling to specify endodermal fate in mouse and human ESCs. Transcription factors that are activated by Nodal signaling include Mix-like homeodomain proteins, Gata zinc finger factors, Sox HMG domain proteins, and Fox forkhead factors [9]. Many genes expressed in DE are also expressed in mesoderm, neuroectoderm, and extraembryonic endoderm (EE). For example,* Foxa2* is expressed in both DE and mesoderm;* Sox 17* is expressed in DE and EE, and hence there is no single marker to identify DE. Nonetheless, the DE population is marked by the coexpression of FOXA2 [10] and SOX17 [11] though individually both of these markers are not specific for DE. Due to heterogeneity in ESC differentiation cultures, the presence of DE markers and the absence of markers of nontarget cell types are used to determine DE-enriched populations. Further differentiation of DE to pancreatic cell types has been reported using a cocktail of growth factors, including FGF10, FGF7, and RA, and inhibitors of key signaling pathways, including Noggin, KAAD-cyclopamine, SANT-1, and Alk5 inhibitor [12–16].

However, current ESC to *β* cell differentiation protocols are limited by low efficiency and generation of immature polyhormonal cells as well as a formation of not-so-robust glucose responsive cells [12, 17–21]. This leads us to believe that some important transcriptional events that are necessary for proper pancreatic development are missing. PTF1a, a critical determinant of pancreatic fate, is not rigorously expressed/is lacking in many of the published differentiation protocols [12, 13, 18, 20, 22]. The significance of the role of PTF1a in committing foregut endodermal cells to pancreatic lineage was elucidated by acquisition of a duodenal fate by pancreatic progenitors that lacked PTF1a in murine transgenic lineage tracing systems [23]. We have previously shown that ectopic expression of PTF1a in mouse ESCs can be used to model pancreas development* in vitro* and results in the generation of monohormonal endocrine cells embedded in exocrine tissue [24]. However, the correct context of PTF1a signaling that is sufficient to direct differentiation to the pancreatic lineage has not been investigated in ESCs until now, and it would be of interest to test if PTF1a signaling can overcome the deficiencies in the current methods of differentiation. In this study, we addressed this question using our* in vitro* model of pancreas development, wherein PTF1a was induced in populations of cells exclusively differentiated to DE or its derivatives.

## 2. Materials and Methods

### 2.1. Cell Lines

The generation and characterization of* Tet-Ptf1a* line that was used in this study are described in [24].

### 2.2. mESC Maintenance and Differentiation

*Tet-Ptf1a* ESCs were maintained in an undifferentiated state on MEF feeder layers with LIF in DMEM-high glucose with 15% FBS, 100 U penicillin/streptomycin, 2 mM L-glut, 2 mM NEAA, 1 mM Sodium pyruvate, and 0.05 mM *β*-mercaptoethanol and cultured in differentiation media as previously described [26], with the following modifications. Media compositions of various differentiation protocols that we tested are enumerated in Table 1. Initial cell seeding density is also indicated for every protocol. The growth factors are abbreviated as Activin A (A), BMP4 (B), and bFGF (F) and their respective concentrations are indicated by the numbers that follow in ng/mL. To induce PTF1a expression, cultures were exposed to 1 *μ*g/mL doxycycline (Dox) renewed every 24 hrs for 3 or 4 days as indicated for the individual experiments. Cells were seeded on two different ECM substrates, Matrigel and Gelatin, for some of the experiments. Methodology for the protocol in Figure 8: Cells were seeded at a density of 60,000 cells/cm^2^ in mESC media containing ROCK Inhibitor (Y-27632) either on Gelatin or on Matrigel. Cells were allowed to attach overnight and DE treatment was started the next day. PTF1a was induced by adding 1 *μ*g/mL Dox for 4 days overlapping with the end of primitive foregut endoderm stage (PF) and pancreatic endoderm stage (PE). Following the PE stage, cultures were further treated with Alk5 inhibitor and Nicotinamide (a maturation factor) for 8 days. The source of the growth factors/inhibitors that were used in these experiments is tabulated in Supplementary Table  3 in Supplementary Material available online at http://dx.doi.org/10.1155/2016/6939438.

### 2.3. Quantitative Real-Time PCR

Cells were harvested at various stages by dissolution and homogenization in 0.5 mL of Buffer RLT (Qiagen), and RNA was isolated and purified using Qiagen RNAeasy Mini kits. QPCR was performed using Applied Biosystems gene expression assays. Assay IDs are given in Supplementary Table  1.* Gapdh* was used as an internal control and the comparative threshold method was used to quantify transcript abundance.

### 2.4. Immunofluorescent Staining

Immunostaining was performed as previously described by Kahan et al. [27]. The antibodies and dilutions are listed in Supplementary Table  2. Secondary antibodies were 488, 568, and 647 Alexa Fluors of anti-goat, anti-mouse, anti-rabbit raised in either goat or donkey. Cells were counterstained with DAPI to mark nuclei. Coverslips with adherent stained cells were mounted on glass slides with Prolong Gold Antifade reagent (Invitrogen). Images were generated using A1R-Si Nikon Confocal or a Zeiss Axiovert 200M microscope.

Immunofluorescent staining was also used to qualitatively measure expression of key transcription factors during differentiation. Flow cytometry was not compatible in many samples involving 3D structures that were hard to digest to single cells for FACS analysis. Hence, it was decided that costaining and manual counting method would be used to quantify all the samples to maintain consistency. After differentiating mESCs using a multitude of protocols, the cultures were costained for Foxa2 and Sox17 at the definitive endoderm stage and for Pdx1 and Nkx6.1 at the pancreatic endoderm stage. Single expressing and coexpressing cells were counted for *N* = 3 biological replicates and tabulated in Table 1. Table 1 also indicates seeding densities, format of culture plates, and presence of clustered or scattered expression of markers.

## 3. Results

### 3.1. Cell Seeding Density Has a Significant Effect on Definitive Endoderm Generation

**Figure 1:**
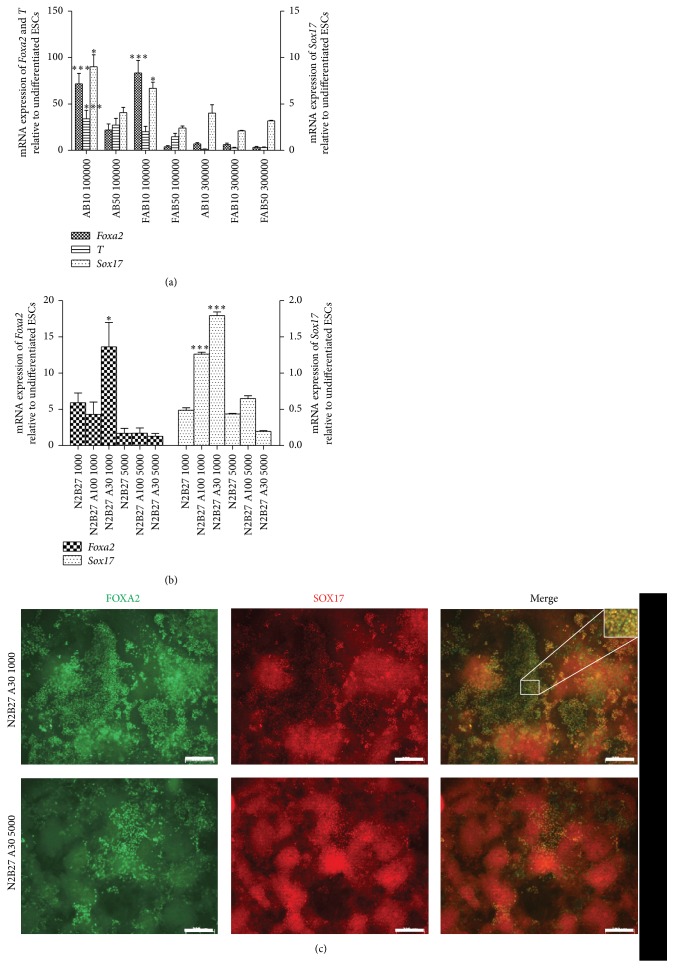
Cell seeding density influences definitive endoderm generation. (a) Cultures seeded with two different mESC densities (100,000 cells/well or 300,000 cells/well of a 24-well plate) were subjected to a 3-day endoderm differentiation protocol containing 100 ng/mL bFGF, 100 ng/mL Activin A, and 10 ng/mL or 50 ng/mL of BMP4. (b) Cells cultured using another endoderm protocol containing Activin A 100 ng/mL or 30 ng/mL for 5 days in N2B27 but seeded with two starting cell densities (1000 cells/well or 5,000 cells/well of a 96 well plate). Relative mRNA expression of* Sox17*,* T*, and* Foxa2* indicates low densities led to higher expression of DE markers. *N* = 2. Data are presented as mean ± SEM. Asterisks indicate *P* values on comparison with corresponding high density cultures: ^*∗*^ *P* < 0.05, ^*∗∗*^ *P* < 0.01, and ^*∗∗∗*^ *P* < 0.001 determined by one-way ANOVA with Tukey's multiple comparison test. (c) Immunofluorescent costaining for FOXA2 and SOX17 confirms that cultures that started with low cell density (N2B27 A30 1000) had higher numbers of FOXA2^+^SOX17^+^ definitive endoderm than cultures seeded with high density (N2B27 A30 5000). Images at 5x. Higher magnification inset to indicate coexpression at the cellular level. Scale bars, 200 *μ*m.

Our published EB-based differentiation protocol that involves culturing mouse ESCs in suspension as embryoid bodies and plating them in 1% SR results in a heterogeneous culture that has only some cells coexpressing SOX17 and FOXA2, two markers of DE (Supplementary Figure 1) [24]. To explore the question of PTF1a induction in an endoderm context, we pursued generating DE efficiently. Numerous DE-induction protocols were tested using growth factors of the TGF-*β* family, including Nodal, Activin A, and BMP4, and small molecules (Table 1). A universal theme among the various protocols was the impact of cell seeding density on differentiation outcome. Low cell densities promoted DE differentiation irrespective of the protocol or the format of the plate used for cell culture. Adaptation of FAB protocol [17], originally devised for hESCs, to mESC differentiation on 24-well plates was more effective in generating DE at an initial cell seeding of 100,000 cells/well versus 300,000 cells/well (corresponding to a cell density of ~50,000 cells/cm^2^ and ~150,000 cells/cm^2^, resp.), as evaluated by* Foxa2*,* T*, and* Sox17* transcripts, and staining for FOXA2 and SOX17 (Table 1). AB10 (Activin 100 ng/mL and BMP4 10 ng/mL) and FAB10 (bFGF 100 ng/mL, Activin 100 ng/mL, and BMP4 10 ng/mL) conditions produced significantly more* Foxa2*,* T*, and* Sox17* transcripts in cultures seeded with 100,000 cells/well than their counterparts seeded with 300,000 cells/well (Figure 1(a)). Similarly, another protocol by Hansson et al. [5] utilizing Activin A in a defined N2B27 medium was more successful in 96-well plates at a lower cell density of 1000 cells/well (~3000 cells/cm^2^) compared with 5000 cells/well (15000 cells/cm^2^). This pattern was, again, observed for two different concentrations of Activin A (Figure 1(b)). Immunofluorescent costaining for FOXA2 and SOX17 was consistent with the transcript profile showing that cultures with low starting cell density formed more DE than high density cultures. Moreover, SOX17^+^FOXA2^+^ cells were always observed at the edges of the colonies (Figure 1(c)) possibly suggesting that DE formation requires less cell-cell contact.

### 3.2. Inducing PTF1a in Monolayer Endodermal-Derived Cells Generated Pdx1^+^Nkx6.1^+^ Pancreatic Progenitors

Achieving DE differentiation from mESC monolayer cultures has been challenging with low yields and poor cell survival.* Tet-Pt1a* mESCs were subjected to several DE differentiation protocols that were published over the course of several years. Initial protocols that were tested had very low efficiency of forming S0X17^**+**^FOXA2^**+**^ cells. Subsequent protocols made modifications to the basal media and additives and/or concentration of growth factors and reported improved DE formation [5, 17], and these also led to robust DE generation by the* Tet-Ptf1a* cells. The results of these protocols are summarized in Table 1. Treatment with Activin A, Nodal, and IDE1 gave rise to DE as marked by SOX17^+^FOXA2^+^ cells (Supplementary Figure 1) and ECADH^+^FOXA2^+^ cells and some ECADH^−^FOXA2^+^ mesodermal cells (Supplementary Figure 2) at differing frequencies. Nodal was the most effective in converting the majority of cells to DE as indicated by the number of SOX17^+^FOXA2^+^ and ECADH^+^FOXA2^+^ cells. Ectopic PTF1a expression was induced in DE obtained from many of these protocols to test the hypothesis that PTF1a activity triggers the pancreatic developmental program more efficiently in an endoderm enriched population compared to spontaneously differentiated EB cultures and ultimately leads to enhanced pancreatic differentiation. Pdx1 activation was designated as the first landmark of pancreatic differentiation, in particular pancreas specification of the naïve endoderm. A short PTF1a induction for 3 days (1 *μ*g/mL Dox addition), either sequentially following DE generation or overlapping with the last 2 days of DE generation, was performed. PDX1 expression was evaluated shortly after (ranging from 3 to 4 days) PTF1a induction. Scattered Pdx1^+^ cells were found in cultures that were grown using the Hansson protocol [5], whereas nodal-generated DE [7] produced Pdx1^+^ bud-like clusters after sequential PTF1a induction (data not shown). However, when these cultures were analyzed a week later, PDX1 expression was absent (results compiled in Table 1). These observations suggested that unpatterned SOX17^+^FOXA2^+^ DE population does not have the right cellular context to respond to PTF1a activity.

**Figure 2:**
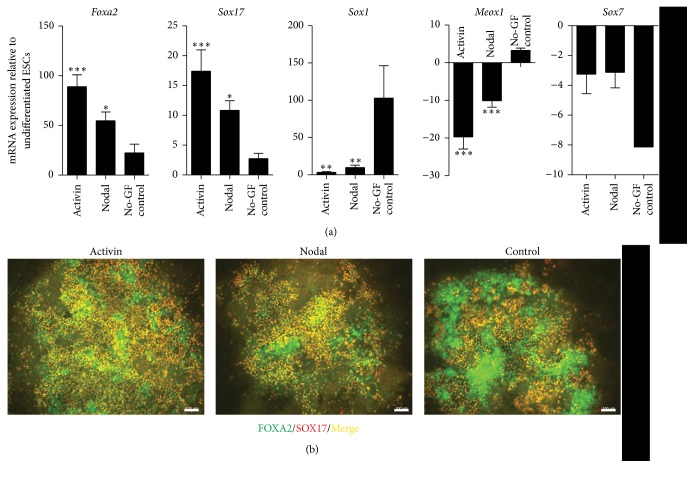
Markers of DE are highly expressed in Nodal and Activin A treated cells, whereas markers of other lineages, including ectoderm, mesoderm, and extraembryonic endoderm are repressed. (a) DE markers,* Sox17* and* Foxa2*, are upregulated in contrast to the genes of other germ layers, ectoderm (*Sox1*), mesoderm (*Meox1*), and extraembryonic endoderm (Sox7) in Nodal and Activin A treated cultures. *N* = 3. Data are presented as mean ± SEM. Asterisks indicate *P* values on comparison with no-growth factor treated (No-GF) control cultures: ^*∗*^ *P* < 0.05, ^*∗∗*^ *P* < 0.01, and ^*∗∗∗*^ *P* < 0.001 determined by one-way ANOVA with Tukey's multiple comparison test. (b) Activin A and Nodal treatment lead to substantial number of FOXA2^+^SOX17^+^ cells, whereas the No-GF control cultures generate FOXA2^+^SOX17^−^ populations. Scale bars, 100 *μ*m.

**Figure 3:**
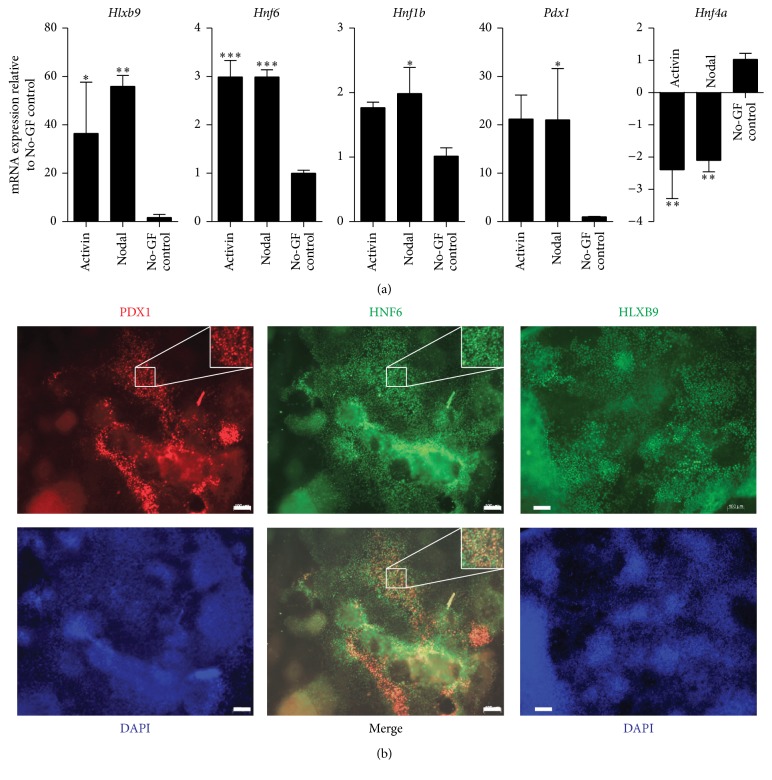
Analysis of mESC-derived posterior foregut endoderm. (a) Posterior foregut endoderm markers,* Hlxb9*,* Hnf6*,* Hnf1b*, and* Pdx1*, were significantly higher in Nodal- and Activin-derived cultures than in no growth factor treated (No-GF) control cultures. On the other hand,* Hnf4a* that is expressed in liver progenitors was reduced. *N* = 2-3. Data are presented as mean ± SEM. Asterisks indicate *P* values on comparison with No-GF control cultures: ^*∗*^ *P* < 0.05, ^*∗∗*^ *P* < 0.01, and ^*∗∗∗*^ *P* < 0.001 determined by one-way ANOVA with Tukey's multiple comparison test. (b) Immunofluorescent staining of Nodal-derived posterior foregut endoderm cultures indicates the expression of the above-mentioned markers at the single-cell protein level. Pdx1 is expressed in Hnf6-expressing cells. Scale bars, 100 *μ*m.

Hence, we pursued further differentiation of naïve endoderm into primitive gut (PG) and posterior foregut endoderm (PF) (modified Melton protocol) to establish the right cell types before inducing PTF1a. Activin A (50 ng/mL) or Nodal (1000 ng/mL) in low serum media was used to generate endoderm. DE markers,* Sox17* and* Foxa2*, were significantly upregulated in both Activin A- and Nodal-DE compared to control cultures (cells that did not receive growth factors but were cultured in the same base media indicating spontaneous differentiation) (Figure 2(a)). Activin A- and Nodal-DE had more SOX17^+^FOXA2^+^ cells than control cultures, and the control cultures also had large numbers of SOX17^−^FOXA2^+^ mesodermal cells (Figure 2(b)). As these two transcription factors are also present in extraembryonic endoderm (EE), it is important to determine the expression of* Sox7*, a marker of EE. Notably,* Sox7* expression was reduced in DE cultures. In addition,* Sox1*, a regulator of ectoderm lineage, and* Meox1*, an indicator of mesoderm differentiation, were significantly lower in Activin A- and Nodal-DE than in controls (Figure 2(a)). After subsequent differentiation to PG and PF, cultures were analyzed for markers of PF. There were significantly higher levels of* Hlxb9* and* Hnf6* transcripts in Activin A-PF (*P* < 0.05 and *P* < 0.001, resp.) and Nodal-PF (*P* < 0.01 and *P* < 0.001, resp.) compared to control cultures that did not receive any growth factors.* Hnf4a*, a liver progenitor maker, was downregulated in both Activin A-PF and Nodal-PF cultures indicating that they have been specified to the pancreatic lineage (*P* < 0.01).* Hnf1B* and* Pdx1* were significantly elevated in the Nodal cultures (*P* < 0.05) (Figure 3(a)). Immunofluorescent staining of Nodal-PF cultures confirms the gene expression profile (Figure 3(b)). PDX1^+^ areas, however, were very few and typically appeared as a subset of HNF6^+^ domains (Figure 3(b)).

**Figure 4:**
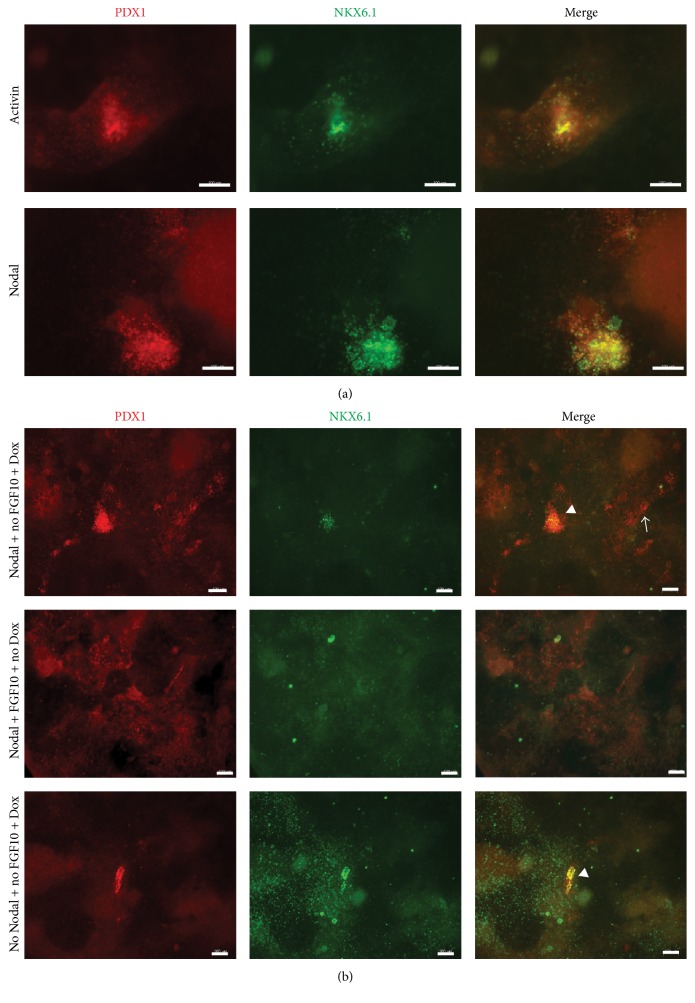
PTF1a induction in combination with FGF10 led to the formation of pancreatic epithelial progenitors. (a) Both Nodal and Activin A derived posterior foregut endoderm generated PDX1^+^NKX6.1^+^ pancreatic progenitors on induction of PTF1a along with FGF10 treatment. Uninduced cultures did not have any such copositive area (data not shown). (b) Nkx6.1 and Pdx1 copositive pancreatic progenitors were unique to PTF1a induced cultures (top and bottom row). FGF10 was not necessary to generate PDX1^+^NKX6.1^+^ population, although it increased the numbers of such progenitors (top row). Notably, FGF10 alone did not produce any NKX6.1^+^ cells (middle row), and hence PTF1a induction is essential for the formation of this pancreatic progenitor population. Arrowheads indicate PDX1 and NKX6.1 double positive costained population. Arrows indicate PDX1 single positive cells. Scale bars, 100 *μ*m.

FGF10 is involved in the proliferation of pancreatic progenitors [29, 30] and in the maintenance of PTF1a expression in the dorsal pancreatic bud [31]. Therefore, we treated the Activin A-PF and Nodal-PF with FGF10 during the induction of PTF1a. PTF1a induction in both Activin A-PF and Nodal-PF cultures resulted in many PDX1^+^NKX6.1^+^ areas. For example, larger domains of PDX1 expression containing small clusters of NKX6.1^+^ cells can be seen (Figure 4(a)). Nkx6.1 is initially expressed in the pancreatic epithelium, but it is a marker of the trunk domain that is poised to become endocrine/duct lineage at later stages. Next, we wanted to investigate whether the formation of pancreatic epithelium was specific to the PTF1a-induced cultures or was caused by FGF10. Nodal-PF cultures that were induced with Dox but not treated with FGF10 also gave rise to PDX1^+^NKX6.1^+^ cells, though lower in number than combined treatment (Figure 4(b), top row). More importantly, Nodal-PF cultures treated with FGF10 alone did not produce any NKX6.1^+^ cells (Figure 4(b), second row). These results suggest that PTF1a induction is important to generate a true pancreatic epithelial progenitor phenotype (PDX1^+^NKX6.1^+^), and that it acts synergistically with FGF10. Above all, it is to be noted that inducing PTF1a in the non-Nodal-PF cultures (i.e., not endodermal enriched cultures) resulted in a few small isolated PDX1^+^ clusters and wide-spread scattered NKX6.1 expression (Figure 4(b), third row) with the PDX1^+^ cells expressing NKX6.1 but with many NKX6.1^+^PDX1^−^ cells as well. These NKX6.1^+^PDX1^−^ cells are not pancreatic and could potentially be of neural lineage, as shown by elevated levels of Sox1 in control (no growth factor treated) cultures early in differentiation (Figure 2(a)). In other words, PTF1a activity in an endodermal context, specifically in prepatterned posterior foregut endoderm, improves the formation of pancreatic progenitor cells compared to induction in spontaneously differentiated cultures.

### 3.3. Differentiation to Adult Pancreatic Cell Types Is Enhanced by 3D Culturing in the DE/PE Stage

**Figure 5:**
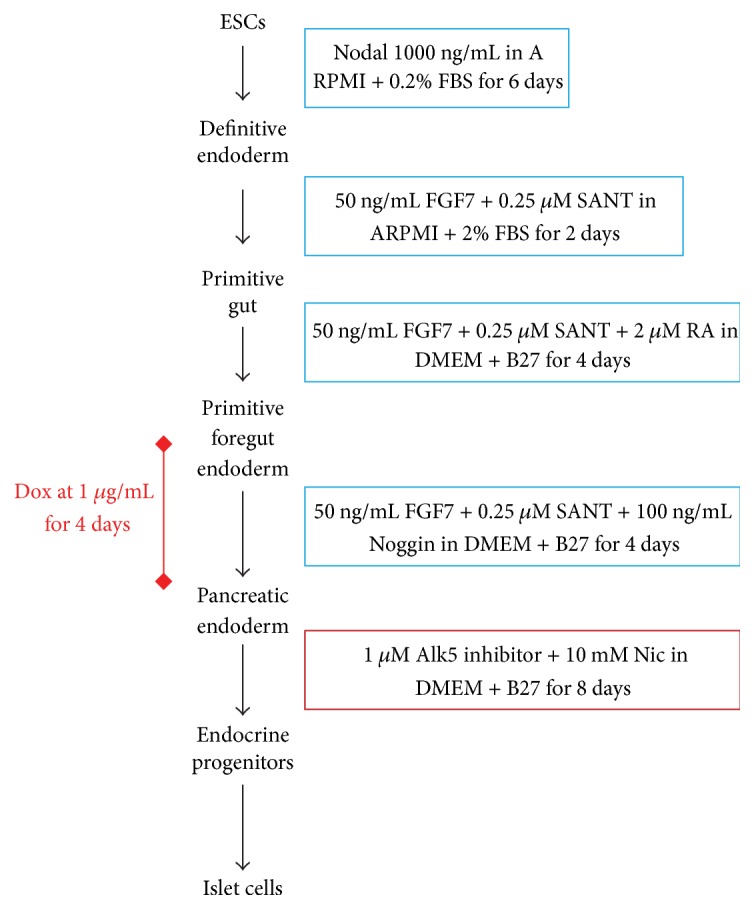
Pancreatic differentiation protocol adapted from Sneddon et al. [25]. Cells were seeded at a density of 60,000 cells/cm^2^ either on Gelatin or on Matrigel. PTF1a was induced by adding 1 *μ*g/mL Dox for 4 days overlapping with the end of primitive foregut endoderm stage (PF) and pancreatic endoderm stage (PE). Following the PE stage, cultures were further treated with Alk5 inhibitor and Nicotinamide (a maturation factor) for 8 days and analyzed for markers specific to adult pancreatic cell types.

On further differentiation of monolayer cultures of PTF1a-induced Activin A/Nodal-PF, we did not observe a concomitant increase in terminally differentiated pancreatic cell types, including insulin^+^, glucagon^+^, somatostatin^+^, or amylase^+^ cells; in fact, only rare insulin^+^ cells were found. Monolayer cultures, though homogenous, lack complex morphogenesis and paracrine signaling present in 3D cultures [32, 33] and hence may not be the optimal way to direct differentiation efficiently into adult cell types. While testing monolayer differentiations on two different ECM substrates, Gelatin or Matrigel, using a protocol adapted from Sneddon et al. [25] (Figure 5), we observed an interesting phenomenon. Floating EB-like subpopulations emerged in both DE and PG stages from Gelatin-coated dishes. These spheres arose only from Gelatin-coated dishes possibly due to differences in integrin signaling mediating cell-ECM interactions on those two substrates. It has been shown that E-cadherin is more stable in cells on Matrigel than on Gelatin resulting in more stable attachment to Matrigel [34]. Serendipitously, the floating spheres were collected, replated, and differentiated as per the protocol till the end stage.

**Figure 6:**
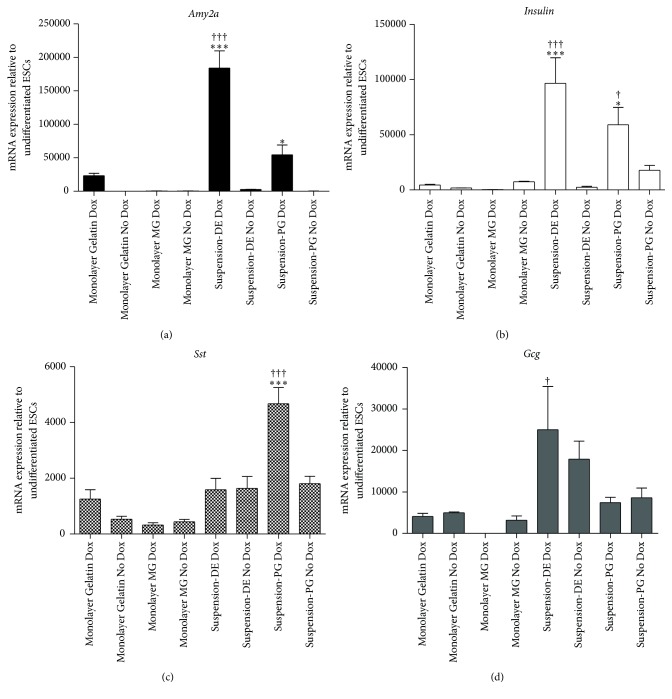
A short 3D culture period at the beginning of differentiation promotes PTF1a-induced pancreatic differentiation. Populations that formed EB-like floating bodies either in DE or in PG stage, when replated and subjected to the differentiation protocol, expressed significantly more* Amy2a* (a),* Insulin* (b),* Sst* (c), and* Gcg* (d) transcripts than those cultures grown as monolayers throughout. Growing in suspension also improved endocrine differentiation in general (Suspension-DE No dox and Suspension-PG No dox) indicated by elevated* Insulin*,* Sst*, and* Gcg* transcripts. *N* = 3. Data are presented as mean ± SEM. Asterisks indicate *P* values on comparison with Gelatin-No Dox cultures: ^*∗*^ *P* < 0.05, ^*∗∗∗*^ *P* < 0.001, and Obelisks indicate *P* values on comparison with Gelatin-Dox cultures: ^†^ *P* < 0.05 and ^†††^ *P* < 0.001 determined by one-way ANOVA with Tukey's multiple comparison test.

It is to be noted that the two ECMs differed in their propensities to generate DE: cells seeded on Gelatin expressed significantly more* Foxa2* than ones seeded on Matrigel, whereas* Sox17* expression was comparable between the two groups (Supplementary Figure 3). PTF1a was induced for 4 days overlapping with the end of PF stage and the beginning of pancreatic endoderm (PE) differentiation as shown in Figure 5. Cells were further cultured for 8 days in Alk5 inhibitor and Nicotinamide, and terminal differentiation to adult pancreatic cell types was assessed. Transcript profiles of endocrine hormones, namely,* Insulin*,* Gcg* and* Sst*, and exocrine digestive enzyme,* Amy2a*, were analyzed in the different cultures. Upon induction of PTF1a, subpopulations that were in suspension (floating) in DE or PG stages (Suspension-DE/Suspension-PG) expressed more endocrine- and exocrine-specific genes than those that were differentiated throughout in adherent cultures (Figure 6). Suspension-DE group had the maximum level of* Insulin*,* Gcg*, and* Amy2a*, whereas Suspension-PE group had maximum* Sst* expression (Figure 6). Cultures that were in suspension for a brief period of time responded better to PTF1a activity and showed enhanced terminal differentiation. Among the adherent cultures, we found that Gelatin performed better than Matrigel as an ECM substrate in the differentiation of Dox-treated cells as judged by significantly higher expression of* Amy2a*,* Gcg*,* Sst*, and* Insulin*. Matrigel, however, seemed to promote the expression of endocrine transcripts in uninduced cultures (Supplementary Figure 4).

**Figure 7:**
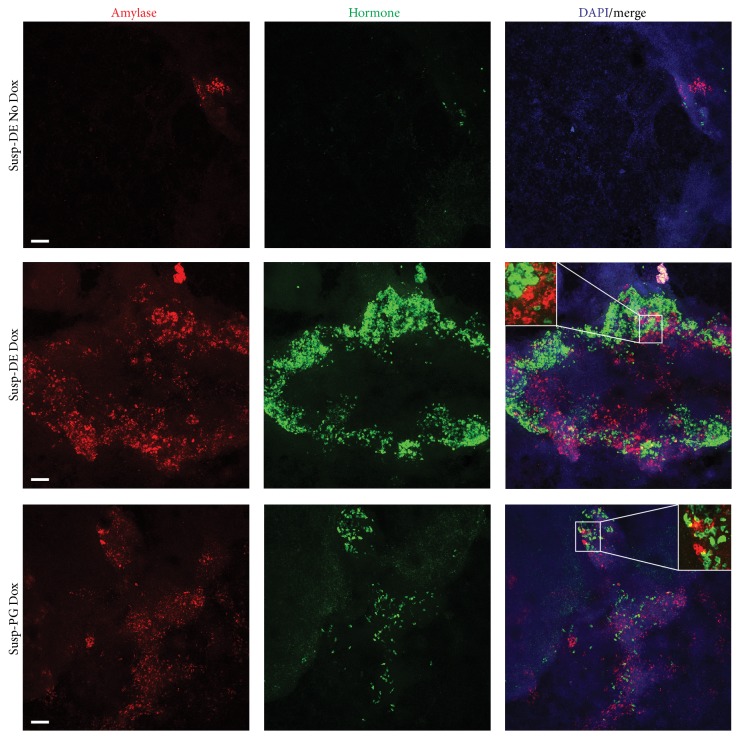
Cultures that were in suspension during either DE or PG stage when induced with PTF1a showed enhanced differentiation to pancreatic cell types. Confocal Z-stacks images at low magnification shown along with DAPI to demonstrate the extent of differentiation between conditions. Susp-DE Dox and Susp-PG Dox cultures had large numbers of Amy^+^ and Horm^+^ cells compared to No Dox cultures and attachment cultures (data not shown). Susp-DE Dox cultures had more Amy^+^ and Horm^+^ cells than Susp-PG Dox cultures. High magnification insets are shown to indicate cytoplasmic staining of hormones and amylase. Scale bars, 50 *μ*m.

**Figure 8:**
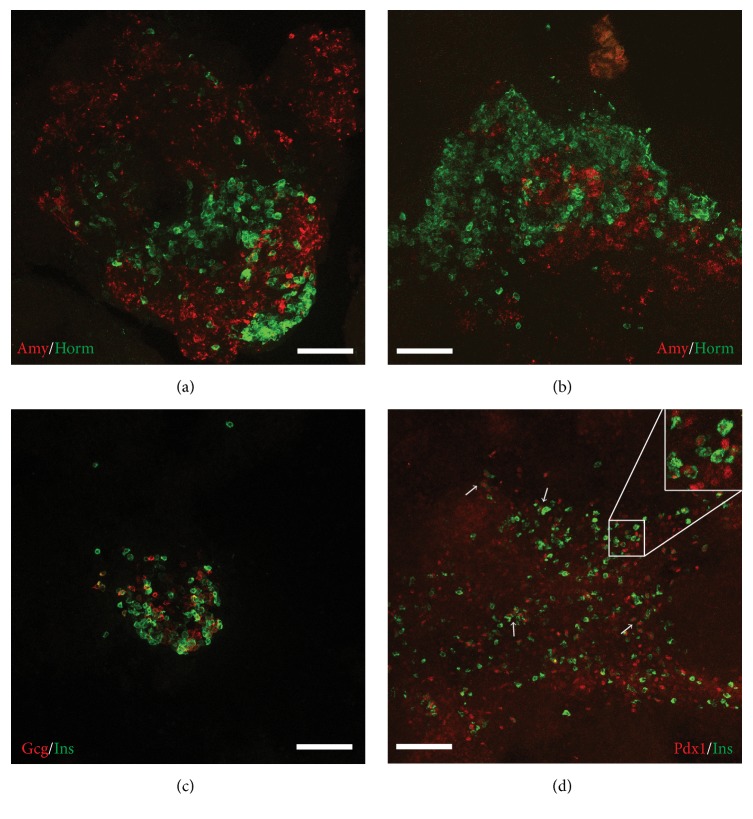
High magnification images of Suspension-DE Dox cultures. (a, b) Horm^+^ and Amy^+^ cells are intermingled with each other. In particular amylase and hormone expression are cytoplasmic and do not overlap. (c) Insulin^+^ cells are mostly monohormonal but comingled with glucagon^+^ cells. (d) Insulin^+^ cells express PDX1 in the nucleus. Inset shows higher magnification to indicate nuclear PDX1 in insulin^+^ cells. Arrows point to many insulin^+^ cell clusters that have nuclear PDX1. Scale bars, 50 *μ*m.

In concert with gene expression, staining for amylase- and hormone-expressing cells (antibody cocktail against insulin, glucagon, and somatostatin) also revealed extensive differentiation of PTF1a-induced Suspension-DE/Suspension-PG cultures to both endocrine and acinar lineage (Figure 7, middle and lower rows). Qualitatively, it appeared that the Suspension-DE cultures produced more endocrine and acinar cells than Suspension-PG cultures. High magnification images of Suspension-DE cultures demonstrated that the amylase^+^ and the hormone^+^ cells were distinct (Figures 8(a) and 8(b)) and that the insulin^+^ cells in the cultures do not express glucagon (Figure 8(c)), while most express nuclear PDX1 (Figure 8(d)).

There was another difference in the way the suspension and the adherent cultures were treated. To enable reattachment, the floating EB-like population was plated in 15% FBS overnight and subjected to subsequent steps in the differentiation protocol as usual. Thus, to rule out that transient serum exposure might be the cause for enhanced pancreatic differentiation, a control experiment was performed where the adherent cultures received overnight serum-bolus mimicking the period of attachment of the floaters. mRNA expression analysis from these cultures (adherent DE/PG) did not show comparable expression of* Amy2a*,* insulin*,* Gcg*, or* Sst* transcripts as the cultures that underwent suspension culturing (Supplementary Figure 5) and immunofluorescent staining (data not shown) confirmed these results, suggesting that the physical state of suspension was responsible for the enhanced pancreatic differentiation that we observed.

## 4. Discussion

The pancreas is an organ of endodermal origin, arising from a narrow* Shh* signaling-excluded region in the posterior foregut endoderm. Therefore, the context of expression of key pancreatic transcription factors (PDX1 and PTF1a) involved in pancreas specification is extremely unique and is important for the successful activation of the pancreatic program. Previously we have shown that ectopic expression of PTF1a in EB-based cultures leads to the formation of all pancreatic lineages: endocrine, acinar, and duct cells. In particular,* in vitro* cultures recapitulated major morphological and molecular events that occur during pancreas organogenesis. In these studies, we hypothesized that induction of PTF1a in an enriched endodermal context would lead to better pancreatic differentiation and that PTF1a could potentially be the missing element in the current *β* cell differentiation protocols that fail to generate monohormonal insulin^+^ cells.

To this end, we generated DE using numerous published protocols as illustrated in Table 1. Many of these protocols showed abysmal performance with low efficiency and/or low cell viability. In contrast to the efficient differentiation of hESC to DE in monolayers using high Activin A and low serum supplementation, mESCs have proven to be rather difficult [1, 3–5, 16]. During our various trials, we observed a common trend: cell seeding density influenced the success of endoderm generation. Cultures that started with low cell densities differentiated to DE more readily than ones with high densities. Our findings correlate well with similar observations that low cell densities promoted RA-induced PDX1 expression in hESCs in culture [35], where cell-cell contact was found to be inhibitory for pancreatic differentiation. It has also been shown that mESCs grown at high densities have their *β*-catenin pool localized at the plasma membrane [36] and membrane association of *β*-catenin with Oct4/E-cadherin is associated with pluripotency [37]. These studies imply that high cell densities may even inhibit differentiation in general. On the contrary, recent reports show contradictory outcomes; high densities promoted pancreatic differentiation [38, 39]. Nonetheless, high/low is a relative term: the seeding density in high density cultures (100,000 cells/cm^2^) in the study by Chetty et al. [39] was midway between low and high density cultures in our experiment (Figure 1(a)) and hence suggests a normal relationship (bell-curve) between seeding density versus DE formation.

Induction of PTF1a in naïve monolayer DE (SOX17^+^FOXA2^+^) populations resulted in only rare Pdx1^+^ cell clusters that were more prominent in Nodal-derived than Activin A-derived DE. It has been suggested than Nodal-derived DE is competent for morphogenesis and organ specification, and hence Nodal is a more relevant molecule for ESC differentiation [6]. But these cells never activated the expression of downstream pancreatic genes, indicating that unpatterned endoderm is not a sufficient context for PTF1a activity. Hence, we differentiated the monolayer DE further to PF and then induced the expression of PTF1a. Importantly, inducing PTF1a in the PF population permitted the generation of PDX1^+^NKX6.1^+^ pancreatic progenitors. FGF10 was found to increase the numbers of PDX1^+^NKX6.1^+^ cells synergistically with PTF1a activity, and FGF10 alone produced isolated PDX1^+^ cells that did not express NKX6.1. Thus, we have demonstrated the necessity of PTF1a expression in the posterior foregut endoderm cells in order to acquire a true pancreatic fate. However, PF-derived PDX1^+^NKX6.1^+^ cells in these cultures did not undergo terminal differentiation. This could be attributed to several factors, one of them being the presence of FGF10 as FGF10 has been shown to force progenitor arrest maintaining them in a proliferative state and abolishes terminal differentiation [30, 40].

While differentiating mESCs in monolayers, we observed some cells spontaneously detaching from the plate and forming floating EB-like populations in suspension especially during the DE and PG stages. Collecting, replating, inducing PTF1a, and differentiating these populations led to considerable adult pancreatic cell types in the cultures, including acinar and hormone-expressing cells. Cells that were in suspension during the DE stage most efficiently generated pancreatic cells. In addition, the insulin^+^ cells that were produced expressed nuclear PDX1 and did not coexpress glucagon suggesting that they represent mature *β* cells. Growing cells in suspension for a short period of time, somehow, enhanced the potential of PTF1a-induced mESCs to progress toward terminal differentiation. 3D aggregates of ESCs or ESC-derived cells possess complex assembly of cellular adhesions, essential for morphogenesis and juxtacrine/paracrine signaling, which are missing in monolayer cultures [32, 33]. Furthermore, additional “inductive” cell type(s) from other germ layers may be generated in 3D cultures that are essential for proper pancreas development. Moreover, EB-based mESC cultures have been differentiated into DE more successfully than monolayer mESCs [2, 41], with maximum DE markers peaking at Day 4. However, EBs also express SHH, an inhibitor of pancreatic fate, from Day 7 [42]. The findings from these studies could be used to explain our results: cells that are in suspension in both Activin A and in low serum differentiate efficiently to DE and are subsequently plated before SHH induction thereby circumventing pancreatic inhibition. Moreover, the plated EB-derived cells are exposed to SANT-1, a SHH signaling inhibitor, in the PG stage reinforcing the elimination of SHH activity.

## 5. Conclusions

In essence, we have demonstrated that PTF1a activity in an endodermal context, specifically in patterned posterior foregut endoderm, improves the formation of pancreatic progenitor cells in mESCs. Additionally, we found that cell density and ECM substrate affect the output of DE and pancreatic cell types. Above all, PTF1a induction in combination with suspension culture formats significantly enhanced differentiation to all adult pancreatic cell types, including insulin^+^ cells that were monohormonal. This study asserts the importance of PTF1a expression in highly enriched endodermal-derived populations to drive the differentiation of ESCs to *β*-like cells efficiently.

## Supplementary Material

Supplemental Information includes 5 figures that corroborate the findings in the article and 3 tables containing information about qPCR primers, antibodies and reagents used in the study.

## Figures and Tables

**Table 1:** Summary of the experimental procedure and results of several endoderm differentiation protocols that were tested on the *Tet-Ptf1a* cells indicating the degree of differentiation to definitive endoderm, Pdx1^+^ cells, and Nkx6.1^+^ cells.

Protocol	Media	Growth factor conc.	Cell seeding number/culture format	Sox17^+^Foxa2^+^ cells	PTF1a induced/days	Pdx1^+^ cells	Pdx1^+^ cells on prolonged culture	
EB in 15% FBS [24]	15% FBS, DMEM for 7 days + 1% SR, DMEM for 2 days	None	3 × 10^6^ cells in 60 mm dish	10%- EB7+2 25%- EB7+4 Not all EBs have copositive cells	Yes. Sequential/3 d	Begin to see at EB7+7	Yes	

Bernardo protocol in monolayer [28]	DMEM (Iscove's modified DM plus Ham's F12 medium at a 1 : 1 ratio), L-Glut, BSA 5 g/L, lipids 1x, 2x ITS, BME 1x.	A100B10 for 3 days A100B10 for 3 days + A100 for 2 days	500,000 per 24 well	Very few and cells unhealthy 5% copositive cells around the edges of colonies	Yes. Sequential/3 days	None 2 clusters (50 cells each)	None None	

	0.5% serum, DMEM, 1% L-glut, 1% penstrep	A100B10 for 3 days		Almost none		None	None	
Low serum		A100B10 for 3 days + A100 for 2 days	500,000 per 24 well	Few cells but culture looks unhealthy	Yes. Sequential/3 days	None	None	
	2% serum, DMEM, 1% L-glut, 1% penstrep	A100B10 for 3 days		Few cells and culture looks little better		None	None	
		A100B10 for 3 days + A100 for 2 days		5–8% copositive cells where density was low		None	None	

Xu protocol w/o Matrigel [17]	DMEM high glucose, 2 g/L BSA, 1% penstrep, 1% L-Glut	A100F100B10	300,000 per 24 well	2-3%	No		None	
		A100F100B50		1-2%	No		None	
		A100B10		1-2%	No		None	
		A100B50		<1%	No		None	
		A100F100B10	100,000 per 24 well	10%	No		None	
		A100F100B50		3-4%	No		None	
		A100B100		10%	No			
		A100B50		5%	No			

Hansson protocol [5]	KO DMEM + 1x-N2 1x-B27 + 1% L-glut + 1% penstrep + Bme + NEAA for 5 days	A100	1000 per 96 well	50–60%	Yes, (a) overlapping (b) sequential/3 d	Yes, (a) scattered cells (b) scattered	Did not see Pdx1^+^ cells on prolonged culture	
		A30		40–50%		Yes, scattered cells		
		A100	5000 per 96 well	10%	No			
		A30		10%	No			
Melton protocol in 96 well [7]	ARPMI + 0.2% FBS + 1% L-glut-penstep	Nodal1000	1000 per 96 well	>90%	Yes, (a) overlapping (b) sequential/3 d	(a) No (b) 15 cell clusters with 20–100 cells each		
		IDE 800 nM		30%		(a) and (b) No		
		IDE 5 *μ*M		High 40–50%	No			
		Nodal1000	5000 per 96 well	>80%	Yes, (a) overlapping (b) sequential/3 d	(a) Scattered cells (b) No		
		IDE 800 nM		>60%		(a) No (b) Scattered cells		
		IDE 5 *μ*M		40–50%	No			

Melton protocol on 24 well	ARPMI + 0.2% FBS + 1% L-glut-penstep	IDE 800 nM	7500 per 24 well	10%	Yes/3 d	None		
			15000 per 24 well	5%	Yes/3 d	None		

		IDE 800 nM		25%	No			
Melton protocol on 24 well w Matrigel	ARPMI + 0.2% FBS + 1% L-glut-penstep on Matrigel	Nodal1000	7500	25%	No			
		A100		1%	No			

Melton modified (obtained from [7])	Definitive endoderm: Activin A (50 ng/mL) or Nodal (1000 ng/mL) in 0.2% FBS in ARPMI for 4–6 days Primitive gut tube: FGF10 (50 ng/mL), KAAD-cyc (0.25 *μ*M) in 2% FBS in ARPMI for 2 days Pancreatic endoderm: FGF10 (50 ng/mL), KAAD-cyc (0.25 *μ*M), RA (2 *μ*M) in B27 DMEM for 4 days PTF1a induction for 4 days in B27 DMEM or 1% SR for Additional culture for 4 more days in same media		2000 per 96 well fed every other day	Activin ~70%. Nodal: 80–90%. After treatment with further factors, some Pdx1 seen in nodal cultures before PTF1a induction	Yes. Sequential/4 d	Activin A: Yes, scattered Pdx1^+^ cells Nodal-yes, Pdx1 in bud-like clusters.	Did not see Pdx1 on culturing for 4 more days	Nkx6.1 expression was seen in some of the Pdx1^+^ cells but these cells did not become insulin^+^ even on ra + nic treatment.

## Abbreviations

1% SR: 1% serum replacement
DE: Definitive endoderm
ECADH: E-cadherin
EB: Embryoid body
FOXA2: Forkhead box protein A2
mESC: Mouse embryonic stem cells
NKX6.1: NK6 homeobox 1
No-GF: No growth factor treated
PDX1: Pancreatic and duodenal homeobox 1
PE: Pancreatic endoderm
PF: Posterior foregut endoderm
PG: Primitive gut
PTF1a: Pancreas specific transcription factor 1a
SOX17: Sex determining region Y-box17
*Tet-Ptf1a*: Tetracycline/doxycycline inducible mouse embryonic stem cell line.
