# Supplementary material for: PTF1a Activity in Enriched Posterior Foregut Endoderm, but Not Definitive Endoderm, Leads to Enhanced Pancreatic Differentiation in an In Vitro Mouse ESC-Based Model
Source: Stem Cells Int. 2016 Jan 6;2016:6939438. doi: 10.1155/2016/6939438 (PMC4811216; doi:10.1155/2016/6939438)
Supplement: Supplementary file 1 — Supplemental Information includes 5 figures that corroborate the findings in the article and 3 tables containing information about qPCR primers, antibodies and reagents used in the study. [file 6939438.f1.pdf]

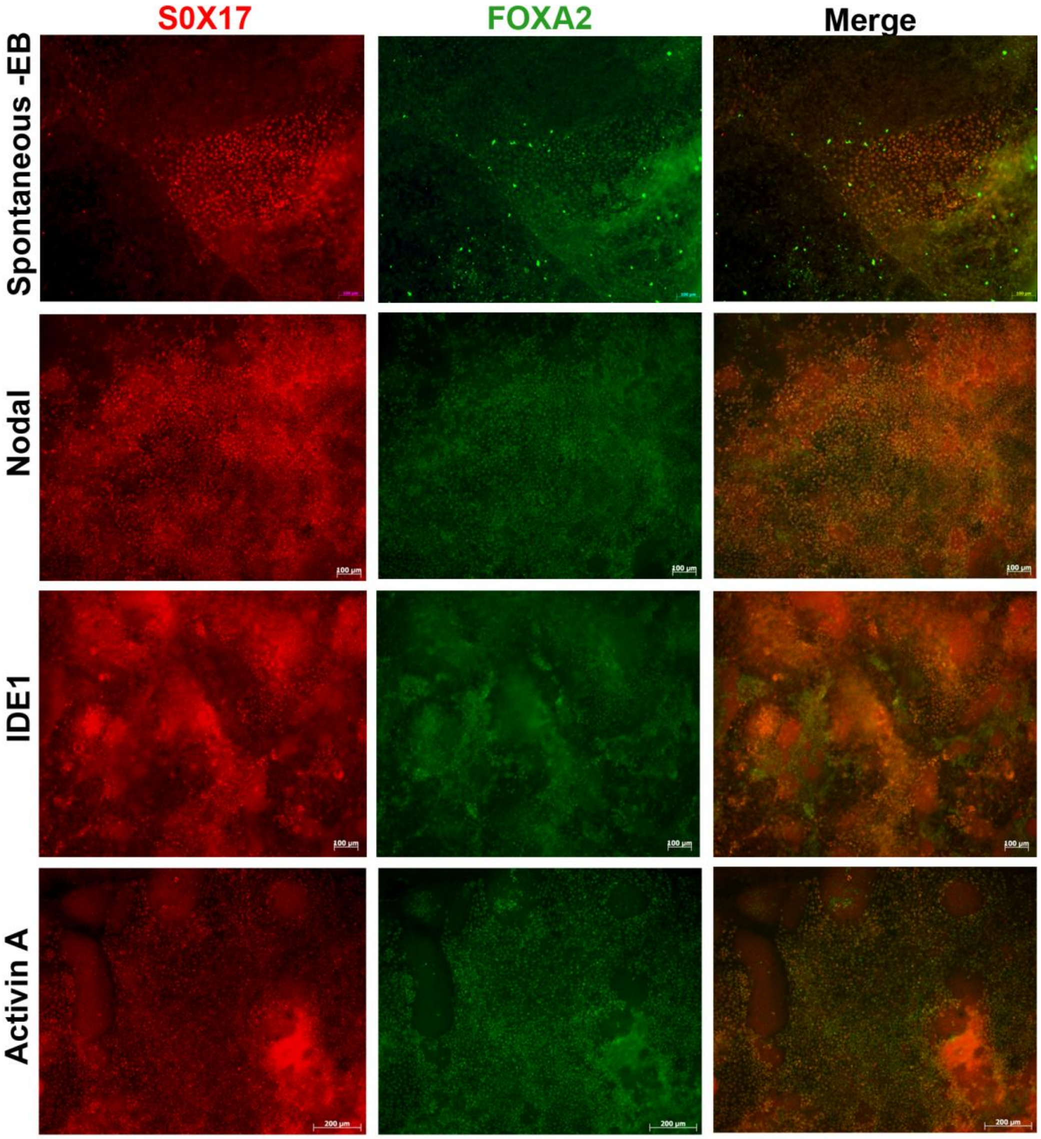

Suppl Fig. 1. Comparison of SOX17 and FOXA2 co-expression after treatment with 1000ng/ml Nodal, 800nM IDE1 0r 100ng/ml Activin. Nodal treatment gave rise to maximum number of $Sox17^{+}Foxa2^{+}$ cells. Scale bars, 100μm.

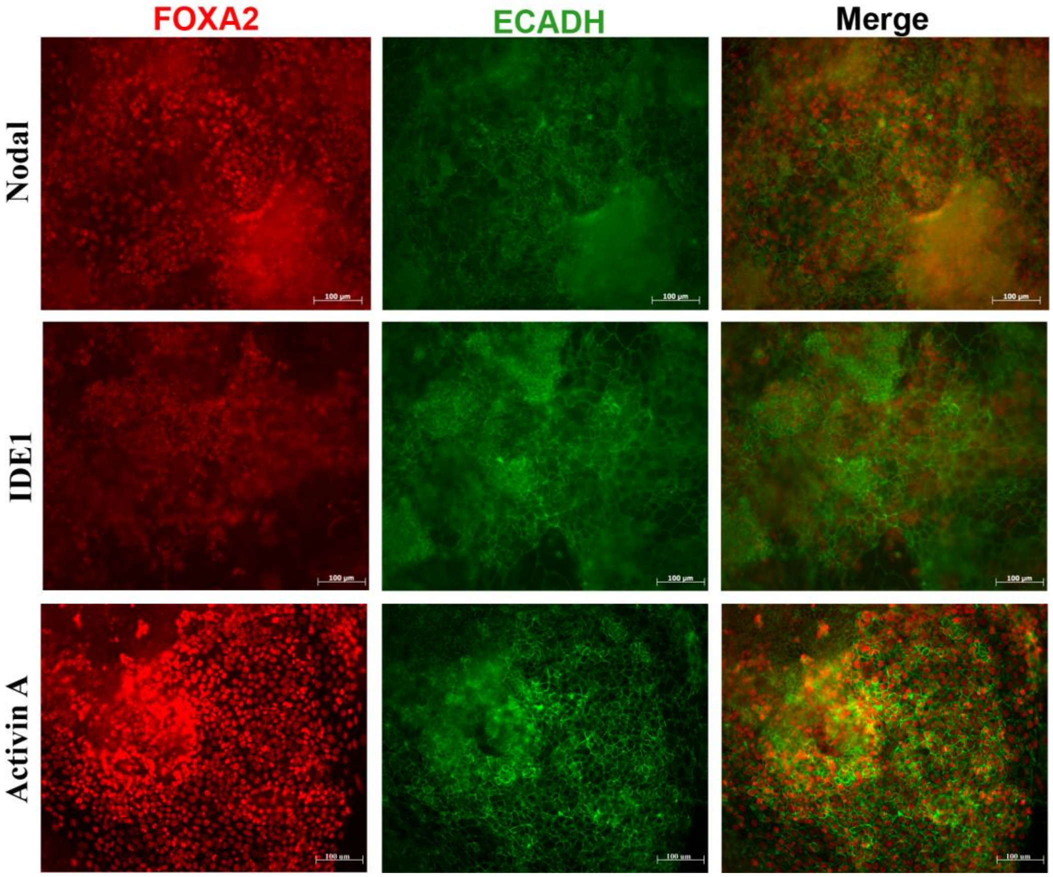

Suppl Fig. 2. FOXA2 and ECADH immunofluorescent staining of cells treated with 1000ng/ml Nodal, 800nM IDE1 or 100ng/ml Activin A. Double positive cells mark definitive endoderm. Scale bars, 100μm.

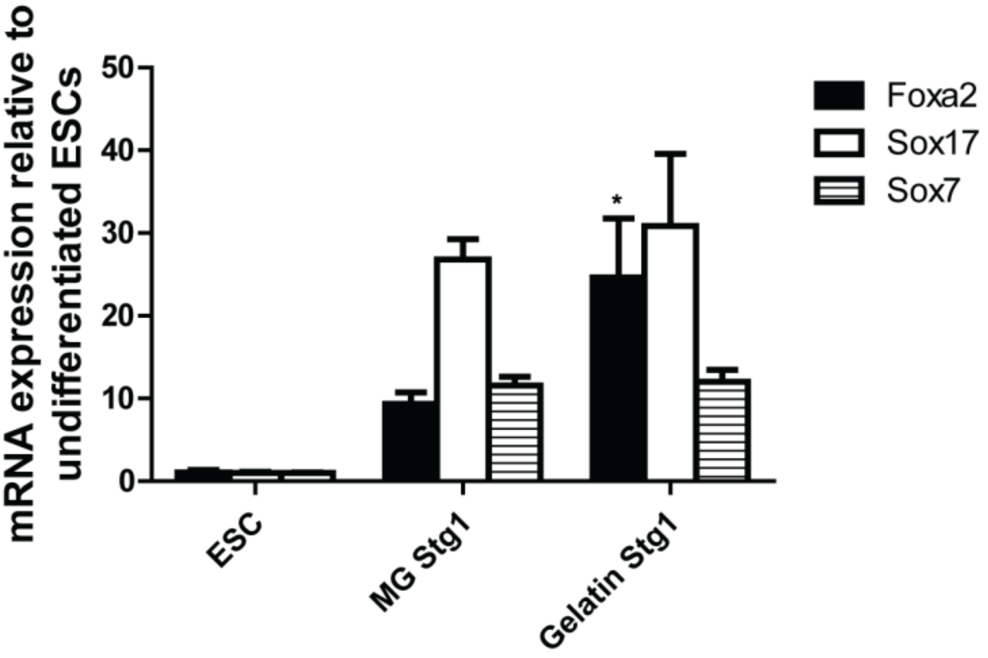

Suppl Fig. 3. Gelatin performed better than Matrigel as an ECM substrate for differentiation of mESC to DE. *Foxa2* transcript levels were significantly higher in cultures grown on Gelatin than Matrigel. Sox17, another DE marker was similarly expressed. N=2. Data are presented as mean ± SEM. Asterisk indicates p values on comparison with Matrigel cultures.*P<0.05 determined by Two-way ANOVA with Bonferroni post- tests.

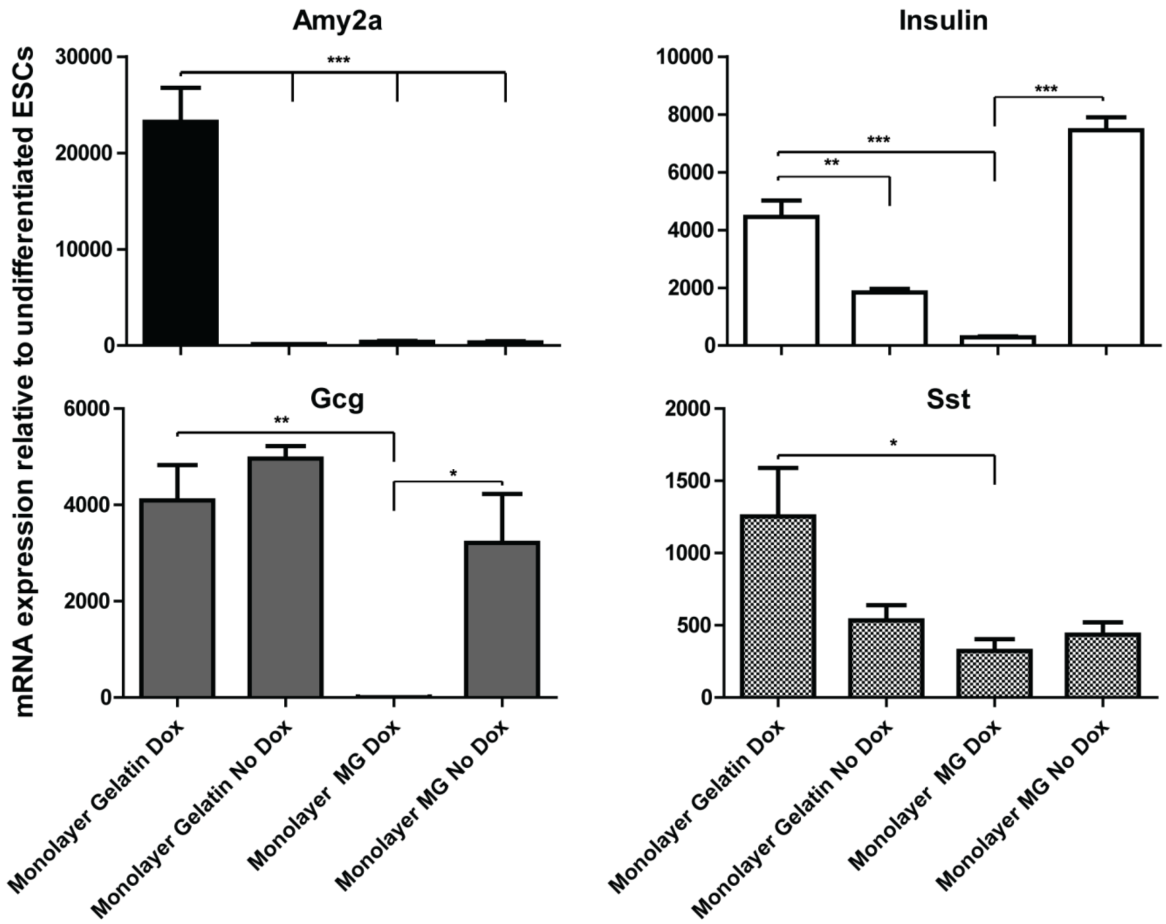

Suppl Fig. 4. Gelatin was more competent than Matrigel as an ECM substrate in aiding pancreatic differentiation of PTF1a-induced monolayer cultures. Dox-induced cultures grown on Gelatin had significantly higher expression of *Amy2a*, *Insulin*, *Sst* and *Gcg* compared to ones on Matrigel. However, endocrine transcripts were elevated in uninduced cultures grown on Matrigel. N=3. Data are presented as mean ± SEM. *P<0.05, **P<0.01, ***P<0.001 determined by One-way ANOVA with Tukey's multiple comparison test.

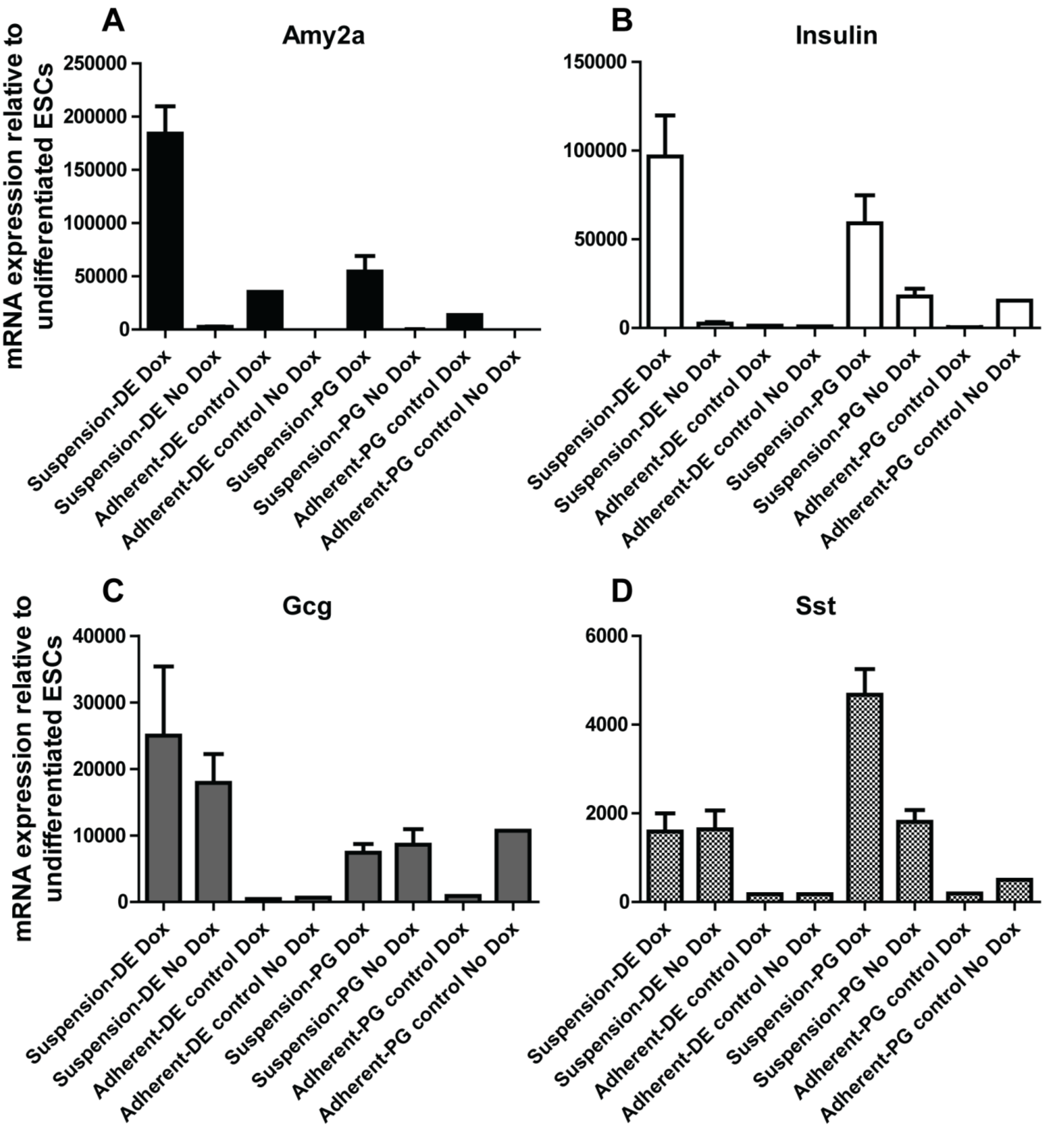

Suppl Fig. 5. The state of suspension is responsible for enhanced differentiation, and not overnight exposure to 15% FBS. Gene expression of pancreatic markers *Amy2a*(A), *Insulin*(B), *Gcg*(C), and *Sst*(D) was not significantly increased in adherent cultures that received an overnight 15%FBS treatment at the same time when the suspension cultures were plated. N=1-3. Data are presented as mean ± SEM.

Supplementary Table 1. List of TaqMan assays

Gene	Assay ID #
Gapdh	Mm99999915_g1
Pdx1	Mm00435565_m1
Ptf1a	Mm00479622_m1
Ngn3	Mm00437606_s1
Glucagon	Mm00801712_m1
Somatostatin	Mm00436671_m1
Amy-2	Mm02342486_mH
Insulin	assay by design MM_INS1-INS1<br>Forward primer GACCCACAAGTGGAACAACTG, Reverse primer AACGCCAAGGTCTGAAGGT, Reporter CTGGGAGGAAGCCCCGGG<br>Custom assay to identify both *Ins-1* and *Ins-2*
Cpa1	Mm00465942_m1
Cel	Mm00486975_m1
Cela3b	Mm00840378_m1
Prss3	Mm02393636_gH
Ctrb	Mm00481616_m1
Iapp	Mm00439403_m1
Chga	Mm00514341_m1
Nkx6.1	Mm00454962_m1
Hes1	Mm01342805_m1
Rbpj	Mm03053645_s1
Rbpjl	Mm00485631_m1
Sox17	Mm00488363_m1
Foxa2	Mm00839704_mH
T	Mm00436877_m1
Sox7	Mm00776876_m1
Sox1	Mm00486299_s1
Hnf6	Mm00839394_m1
Hlxb9	Mm00658300_g1
Hnf4a	Mm01247712_m1
Hnf1b	Mm00447459_m1
Meox1	Mm00440285_m1

Supplementary Table 2. List of primary antibodies for Immunofluorescence

Antibody name	Clone or catalog #	Dilution used	Source
**Primary antibodies**			
Rabbit anti-Foxa2	AB40874	1:2000	Abcam
Rabbit anti-Hnf6	sc-13050	1:250	SantaCruz Biotech
Rabbit anti-Pdx1	na	1:4000	C. Wright, Vanderbilt U
Goat anti-Pdx1	na	1:1000	C. Wright, Vanderbilt U
Rabbit anti-Hnf4α	sc-8987	1:250	SantaCruz Biotech
Rabbit anti-Sox9	AB5535	1:500	Millipore
Rabbit anti-Ptf1a	na	1:4000	Ray Mcdonald, UT
Mouse anti-Nkx6.1	ab2023	1:2000	BCBC
Mouse anti-Ngn3	ab2013	1:2000	BCBC
Mouse anti-Glucagon	K79bB10	1:2000	Sigma
Mouse anti-Insulin	K36aC10	1:2000	Sigma
Rabbit anti-Somatostatin	20067	1:2000	ImmunoStar
Mouse anti-Somatostatin	SOM-018	1:2000	Genway Biotech
Rabbit anti-C-peptide	657	1:2000	BCBC
Rabbit anti-mouse-alpha amylase	na	1:2000	Axell, Westbury
Rabbit anti-mouse amylase	A8273	1:2000	Sigma
Rabbit anti-Cpa	na	1:1000	AbD Serotec
FITC conjugated anti-DBA	FL 1031	1:500	Vector labs
Rabbit anti-Mnx1/Hlxb9	na	1:8000	Samuel L. Pfaff, Salk
Rabbit anti-Hnf1b	sc-7411	1:200	SantaCruz Biotech

na, not available
BCBC, Beta Cell Biology Consortium, www.bcbc.org

Supplementary Table 3. List of growth factors/small molecules used in differentiation of ESCs

Reagent name	Clone or catalog #	Source
Nodal	1315-ND-025	R&D
Activin A	338-AC-050	R&D
BMP4	314-BP-050	R&D
FGF7	251-KG-050	R&D
FGF10	6224-FG-025	R&D
Noggin	6057-NG-100	R&D
Matrigel	na	WiCell
FBS	na	WiCell
bFGF	na	WiCell
GlutaMax	35050061	Life Technologies
NEAA	11140050	Life Technologies
DMEM,High Glucose	11965118	Life Technologies
Advanced RPMI	12633012	Life Technologies
Pen-strep	15140122	Life Technologies
B-27 Serum-free Supplement	17504044	Life Technologies
KO Serum Replacement	10828028	Life Technologies
N2 Supplement	17502048	Life Technologies
Retinoic Acid	R2625-50mg	Sigma
SANT-1	S4572-5MG	Sigma
BSA	A7030-10G	Sigma
Nicotinamide	N0636-100G	Sigma
LIF	5057119	Millipore
ALK5 Inhibitor 2	ALX-270-445-M001	Axxora
IDE1	04-0026	Stemgent

na, not available
